# Skin cancer prevention practices of barbers for their customers: a pilot study

**DOI:** 10.1093/skinhd/vzag079

**Published:** 2026-06-09

**Authors:** Helen Fleming, Chris Wells, Rebecca Stores

**Affiliations:** Faculty of Science and Health, School of Dental, Health and Care Professions, University of Portsmouth, Portsmouth, UK; Faculty of Science and Health, School of Dental, Health and Care Professions, University of Portsmouth, Portsmouth, UK; Faculty of Science and Health, School of Dental, Health and Care Professions, University of Portsmouth, Portsmouth, UK

## Abstract

**Background:**

Most skin cancers develop in the head-and-neck area due to sun-related ultraviolet (UV) damage to unprotected skin and are preventable. Currently, one in four men in the UK will develop skin cancer in their lifetime. Those with short hair have an increased risk of UV damage. Barbers who cut men’s hair could help influence their customers’ head-and-neck skin cancer prevention behaviours.

**Objectives:**

To investigate a sample of UK barbers’ current personal and customer-related skin cancer prevention practices, and practices they may consider using in the future, and to explore influences on customer skin cancer prevention practices.

**Methods:**

Stratified random sampling using Google Maps was used to select a sample of barbers from a UK city who were visited in person and invited to complete a paper survey, which were collected 1–2 weeks later.

**Results:**

Twenty-three of 31 invited participants completed the survey, giving a 74% response rate. Fifty-seven per cent (*n* = 13) reported regularly or always wearing sunscreen themselves, 17% (*n* = 4) reported regularly or always discussing sunscreen with customers, 9% (*n* = 2) reported applying sunscreen to customers and none sold sunscreen. Fifty-five per cent (*n* = 12) indicated they would consider talking to customers about sunscreen, 26% (*n* = 6) would consider applying sunscreen to customers and 35% (*n* = 8) would consider selling sunscreen. Participants’ self-application of sunscreen protection and believing sunscreen protection was important had a statistically significant association with talking to customers about sunscreen, considering talking to customers about sunscreen and considering selling sunscreen.

**Conclusions:**

Most participants reported that they would consider talking to customers about sunscreen protection, over a quarter would consider applying sunscreen protection and over a third would consider selling sunscreen. Barbers could help customers adopt healthier skin cancer prevention behaviours, lowering customers’ risk of developing head-and-neck skin cancers.

## Teaser

Most skin cancers (up to 90%) are preventable and primarily attributable to ultraviolet (UV) radiation. Over 80% arise in the head-and-neck region, reflecting the chronic cumulative effect of UV exposure. In the UK, men suffer a disproportionately high burden, with a lifetime risk of 1 in 4 for nonmelanoma skin cancer and 1 in 35 for melanoma. Men are almost twice as likely as women to develop a head-and-neck melanoma and 69% more likely to die from skin cancer. Studies show that men in the UK are significantly less likely to wear sunscreen than women, despite being at increased risk of cumulative UV exposure through outdoor occupations and hobbies. Improving head-and-neck sunscreen use in men could reduce the risk of skin cancer. Barbershops may represent an untapped opportunity for intervention, by promoting sunscreen use and facilitating access to sunscreen products. Barbers could play a key role in skin cancer prevention strategies targeting high-risk customers.

What is already known about this topic?

- UK studies have previously used barbershops to convey health messages, encourage cancer screening and conduct health monitoring services such as weight, blood pressure and cholesterol checks in hard-to-reach areas of the community.
- Some UK barbers have undertaken formal training to support their customers’ mental health and others training to monitor customers’ health, including blood pressure checks.
- Skin cancer prevention practices of UK barbers for their customers are currently under-researched.

What does this study add?

- Most barbers believe head-and-neck sunscreen protection is important and report regularly wearing sunscreen themselves.
- Only 17% of barbers regularly discuss sunscreen protection with customers.
- Barbers’ positive behaviours and attitudes to sunscreen are significantly associated with discussing sunscreen with customers.
- Most barbers indicate they would consider talking to customers about sunscreen.
- Barbers could become advocates for improving customers’ sun-protection behaviours, potentially lowering their customers’ risk of developing skin cancer.

Skin cancer is the most common cancer in the UK.^1^ In the last decade, rates of nonmelanoma and melanoma skin cancer have risen by 42% and 31%, respectively,^2,3^ with this trend expected to continue. The cost of skin cancer treatment to the National Health Service (NHS) in 2025 was estimated to be £338–465 million.^4^ The continued escalation in the incidence of this disease and its associated treatment cost is likely to become unsustainable.

Skin cancer is highly preventable; almost 9 in 10 cases are caused by ultraviolet (UV) radiation from the sun, which damages the DNA of skin cells causing mutations that can develop into skin cancer.^5^ Over 80% of skin cancers occur in the head-and-neck area due to frequent sun exposure damaging unprotected skin.^6^ Good sun-protection behaviours, including regularly wearing sunscreen on the head-and-neck area, are essential to reducing the risk of UV-related skin damage.

The incidence of skin cancer and mortality statistics in the UK reveal a sex disparity. One in four men will develop a non­melanoma skin cancer compared with one in six women.^2^ In the UK, in the last decade, the incidence of melanoma has increased by 36% for men compared with 25% for women, and 1 in 35 men will develop a melanoma compared with 1 in 41 women.^3^ Melanomas that develop in the head-and-neck area have a worse prognosis than those that develop elsewhere,^7^ with men almost twice as likely as women to develop melanoma in this area.^3^ Men are 69% more likely to die from skin cancer than women,^8^ often presenting to their general practitioner at a more advanced stage of the disease.^9^

One possible contributing cause of this inequality is that men in the UK have been found to be less inclined to wear sunscreen than women. A study conducted in 2022 by Nivea Sun in collaboration with Cancer Research UK surveyed 2000 men in the UK on their sun-safety behaviours and found that although 84% were aware that sunburn caused skin cancer, <25% regularly wore sunscreen. The reasons men gave for not wearing sunscreen were that they thought the sun was not strong enough (25%) and that they did not really think about it (23%).^8^ Similarly, a survey conducted in 2023 on behalf of Melanoma Focus of 2003 UK adults found that 32% of British adults rarely or never used sunscreen, rising to 40% for men, and that only 11% of British adults always wore sunscreen, dropping to 8% for men.^10^ These findings are concerning. Many men suffer an increased risk of cumulative exposure to the sun due to their outdoor occupations and hobbies, and tendency to have short or thinning hair, exposing more skin in the head-and-neck area to potential UV damage. Identifying ways to influence healthier male head-and-neck sun-protection behaviours could potentially reduce the probability of skin cancer occurring in this area.

UK barbershops have previously been recognized as platforms for delivering health messages and interventions to men who may not access healthcare through more traditional routes. Barbershops in Bradford were used in a study as a setting for health workers to carry out checks on customers’ blood pressure, body mass index, blood sugar and cholesterol, and to provide advice on stopping smoking. The study found that men were interested in their health and were more likely to access health services for screening and preventative care if it was made available at a convenient time and place.^11^ Barbershops have also been identified as platforms to encourage men at risk of developing prostate cancer to get themselves checked. Participating establishments were provided with window stickers, decals and leaflets incorporating QR codes linked to prostate cancer risk assessments and advice.^12^ Although there are no formal outcome data for this initiative, run in collaboration with Macmillan Cancer Support, the scheme is still active.

UK barbers have previously been used to support their customers’ mental health via several campaigns.^13–15^ One such initiative, BarberTalk, trained barbers to spot the signs of men struggling with their mental health, to provide supportive listening and to signpost customers to further help. The campaign was set up by a barber whose friend had taken his own life.^16^ Another study that investigated the impact of the COVID-19 pandemic on men’s mental health through their barber revealed barbers’ willingness to undertake training to formalize supporting their customers’ mental health.^17^

Supporting the physical wellbeing of customers of barbers was the subject of a London-based study in which barbers were trained to educate, monitor and support customers at risk of high blood pressure. The study concluded that barbers could be effectively trained to give important healthcare advice on blood pressure and to take blood pressure readings.^18^

We have previously investigated the role of UK barbers and hairdressers in screening customers for skin cancer.^19^ Our study found that although only 5% of participants had received skin-cancer awareness training, almost all (92%) were interested in learning more about skin cancer signs and symptoms and would consider screening customers with training. These studies demonstrate that UK barbers are potentially willing to take action to support their customers’ health.

Although the skin cancer screening practices of UK hairdressers and barbers in their customers has previously been investigated by us, a recent systematic literature review conducted by H.F. found no studies establishing skin cancer prevention practices by UK barbers, either personally or for their customers. To address this gap in knowledge, we conducted a preliminary investigation via a survey to determine the current skin cancer prevention practices of a small sample of UK barbers.

Our survey focused on the use of sunscreens in response to studies conducted by Cancer Research UK and Melanoma Focus concerning poor sunscreen use by men in the UK.^8,10^ Sun-protection measures, such as reducing sun exposure time, using umbrellas or wearing hats or sunglasses, were not explored in this study; however, these measures are most effective when used in conjunction with sunscreen.^20–22^ We also hypothesized that sunscreen use was an area in which barbers might influence head-and-neck skin-protection behaviours in their customers. Some barbers recommend and sell grooming products; therefore, advising on and recommending the use of sunscreen products could be viewed as a natural extension of this service. Skin cancer monitoring habits were also not explored in this study as these practices were investigated in a previous study;^19^ however, these could be looked at in further studies.

## Materials and methods

### Study objectives

The primary objective of this study was to determine barbers’ attitudes and behaviours towards sun protection and the extent to which they were, or would consider, talking to their customers about sunscreen use; were, or would consider, applying sunscreen to customers after a short haircut; or who sold, or would consider selling, sunscreen. Secondary objectives were to analyse influencing factors (e.g. personal sunscreen behaviours and attitudes, participants’ age, sex, ethnicity, years worked as a barber or knowing someone who has had skin cancer) on talking about sunscreen, applying sunscreen or selling sunscreen.

### Study recruitment

A stratified random sampling method was used to recruit barbers from Portsmouth, UK, to complete a paper survey. Portsmouth was chosen as the city in which to conduct the study as it has a 35% higher rate of melanoma incidence than the average for England.^23^ The reason for the city’s high incidence of melanoma has not been established. Traditional barbershops representing each postcode area of the city from PO1 to PO6 were identified and randomly selected using Google Maps. Participants were eligible to complete the survey if they worked in one of the randomly selected barbershops. We aimed to recruit 4 barbers for each postcode area, totalling 24, to ensure a socioeconomically mixed representation of the city. This number was also considered achievable while adhering to the design criteria. The study intended to recruit barbers working in traditional barbershops with a predominantly male customer base. A maximum of two barbers were permitted to participate per establishment, to ensure a wide diversity of views for each postcode area. The first author (H.F.) visited barbers in person in these establishments, explained the research study and invited them to complete the survey. Completed surveys were collected in person by H.F. the following week.

### Survey

A paper-based survey method was selected based on our prior research study, as this design had previously yielded a high response rate among hair professionals.^19^ The 17-question paper survey included questions on participants’ demographics and 5-point Likert scale questions that investigated participants’ personal sunscreen attitudes and behaviours, current sunscreen practices for customers and their willingness to consider providing customer sun-protection services in the future. An open question was included that allowed participants to add their own thoughts on providing skin cancer prevention services to customers. Four barbers were purposefully chosen outside of the study area to pilot the participant information sheet and survey to ensure its clarity, conciseness and comprehensibility, and that the survey questions were unambiguous and relevant.

### Statistical analysis

Data were analysed using SPSS 28 (IBM, Armonk, NY, USA). Descriptive statistics were used to describe participants’ demographics. A nonparametric analysis method was chosen to determine associations between ordinal and nominal data to allow for the variation in data distribution and the small sample size. Spearman’s rank correlation was used to explore any significant associations between participants’ personal sunscreen attitudes and behaviours, and their customers’ sunscreen attitudes and behaviours, and to identify any relationship between age or time in occupation and customers’ sunscreen attitudes and behaviours. Pearson’s χ^2^ test was used to identify any significant influencing factors on customer sunscreen behaviours such as sex or knowing someone who has had skin cancer. A two-tailed *P*-value <0.05 was considered statistically significant.

## Results

### Participants

Nineteen barbershops were approached and 31 barbers invited to participate; surveys were completed by 23 participants from 14 establishments (a 74% response rate, *n* = 23) to achieve equal representation of the city’s postcode areas.

Ninety-one per cent (*n* = 21/23) of the participants were men [mean (SD) age 41.0 (12.0) years]. Participants had a mean (SD) duration in the occupation of 14.96 (12.93) years. Of those who reported their ethnicity, most identified as British (*n* = 13; 57%) or English (*n* = 3; 13%) (Table 1).

**Table 1:** General characteristics and demographics of the participants (*n* = 23)

Characteristic/demographic	*n* (%)
Age (years), mean (SD)	41.0 (12.0)
Sex	
Male	21 (91)
Female	2 (9)
Ethnicity	
British	13 (57)
English	3 (13)
Unknown	3 (13)
Iraqi	2 (9)
Mixed British	1 (4)
Kurdish	1 (4)
Time in occupation (years), mean (SD)	14.96 (12.93)

### Participants’ personal sun-protection behaviours and attitudes

Sixty-one per cent of participants (*n* = 14) knew about sunscreen products for the head-and-neck area, 57% (*n* = 13) indicated they frequently or always applied sunscreen products to their head-and-neck area and 57% (*n* = 13) thought it very important to wear head-and-neck sunscreen protection to prevent sunburn. No participants reported they had previously had skin cancer, but 39% (*n* = 9) knew someone who had (Table 2).

**Table 2:** Participants’ personal sunscreen behaviours and attitudes (*n* = 23)

Survey question	*n* (%)
Do you know about sunscreen products for the head-and-neck area?	
Yes	14 (61)
No	0 (0)
Somewhat	8 (35)
Don’t know	1 (4)
Do you apply sunscreen products to your head-and-neck area?	
Never	2 (9)
Rarely	3 (13)
Sometimes	5 (22)
Frequently	10 (43)
Always	3 (13)
How important do you think it is to wear head-and-neck sunscreen protection to prevent sunburn?	
Not at all important	0 (0)
Low importance	0 (0)
Don’t know	4 (17)
Moderately important	6 (26)
Very important	13 (57)
Have you had skin cancer?	
Yes	0 (0)
No	23 (100)
Prefer not to say	0 (0)
Do you know someone who has had skin cancer?	
Yes	9 (39)
No	13 (57)
Prefer not to say	1 (4)

### Participant-reported current customer sun-protection behaviours and future behaviours

Seventeen per cent of participants (*n* = 4) reported that they frequently or always talked to customers about using sunscreen to protect the head-and-neck area, 9% (*n* = 2) sometimes applied sunscreen to customers after giving them a short haircut, 52% (*n* = 12) thought it somewhat or very unlikely that their customers would like them to apply sunscreen protection to their head-and-neck area after a short haircut and none of the participants sold sunscreen protection in their barbershop (Table 3).

**Table 3:** Participants’ current sunscreen behaviours with regard to customers and planned future sunscreen behaviours (*n* = 23)

Survey question	*n* (%)
Do you talk about sunscreen protection in the head and neck area with your customers?	
Never	6 (26)
Rarely	7 (30)
Sometimes	6 (26)
Frequently	3 (13)
Always	1 (4)
Do you apply sunscreen protection to the head-and-neck area of your customers after giving them a short haircut?	
Never	21 (91)
Rarely	0 (0)
Sometimes	2 (9)
Frequently	0 (0)
Always	0 (0)
In your opinion, how likely is it that your customers would like you to apply sunscreen protection to their head-and-neck area after a short haircut?	
Very unlikely	8 (35)
Somewhat unlikely	4 (17)
Neither likely nor unlikely	5 (22)
Somewhat likely	2 (9)
Very likely	3 (13)
Don’t know	1 (4)
Do you sell sunscreen products in your shop?	
Yes	0 (0)
No	23 (100)
Don’t know	0 (0)
How likely is it that you would consider talking about sunscreen protection with your customers after a short haircut?^a^	
Very unlikely	3 (14)
Somewhat unlikely	3 (14)
Neither likely nor unlikely	4 (18)
Somewhat likely	7 (32)
Very likely	5 (23)
Don’t know	0 (0)
How likely is it that you would think about applying sunscreen to your customers head-and-neck area after a short haircut?	
Very unlikely	8 (35)
Somewhat unlikely	5 (22)
Neither likely nor unlikely	4 (17)
Somewhat likely	3 (13)
Very likely	3 (13)
Don’t know	0 (0)
How likely is it that you would consider selling sunscreen products in your shop?	
Very unlikely	3 (13)
Somewhat unlikely	2 (9)
Neither likely nor unlikely	9 (39)
Somewhat likely	3 (13)
Very likely	5 (22)
Don’t know	1 (4)

^a^Where numbers do not equal *n*, there are missing data as not all questions were responded to.

Over half (*n* = 12; 55%) of participants who responded thought it somewhat or very likely that they would consider talking to customers about sunscreen protection after a short haircut, 26% (*n* = 6) thought it somewhat or very likely that they would consider applying sunscreen protection to customers after a short haircut and 35% (*n* = 8) thought it somewhat or very likely that they would consider selling sunscreen protection in their barbershop. Figure 1 shows the percentage of participants currently talking about skin cancer prevention with customers and those who would consider talking about skin cancer prevention with customers in the future.

**Figure 1:**
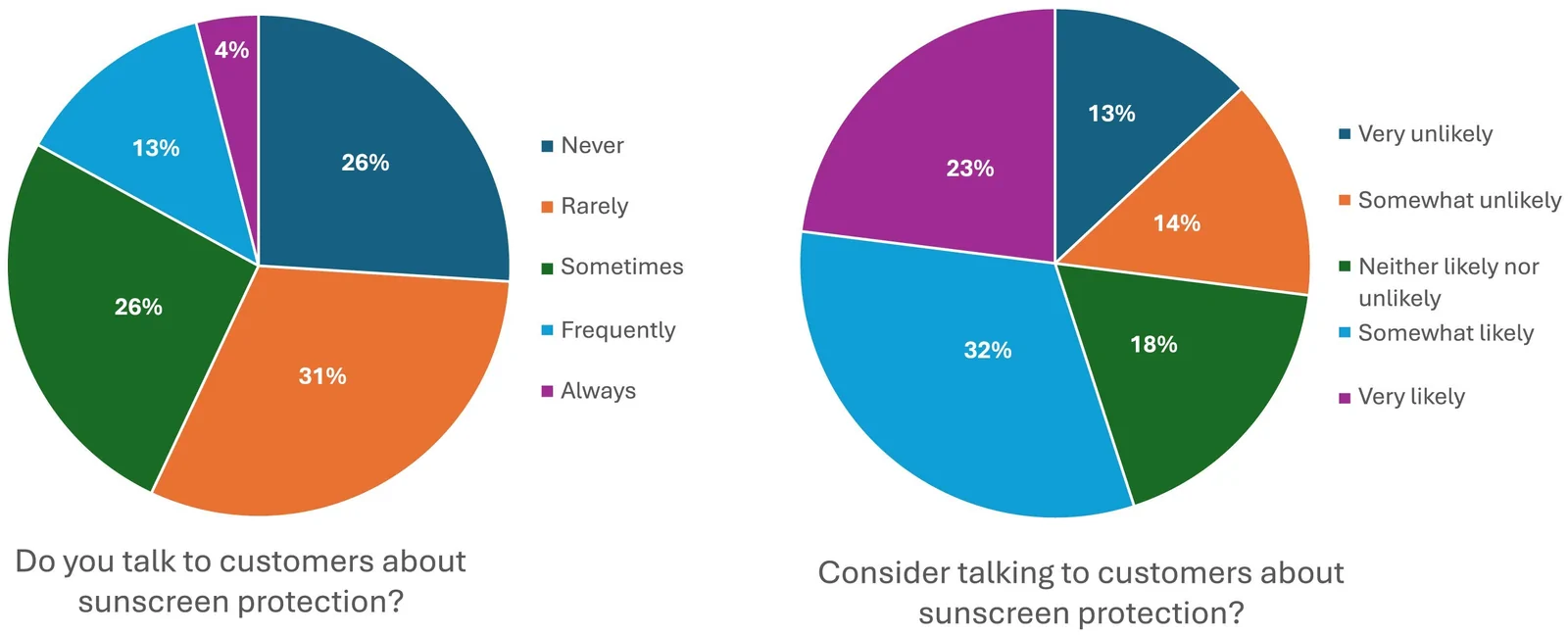
Participants who currently talk to customers about sunscreen and those who would consider talking to customers about sunscreen in the future.

### Participants reported personal and customer sunscreen use

Spearman’s rank correlation was used to determine significant associations between participants’ reported personal and customer sun-protection behaviours. Responses were measured using a Likert scale, where 1 = never, 2 = rarely, 3 = sometimes, 4 = frequently and 5 = always. A statistically significant positive correlation was found between participants’ self-application of sunscreen and their frequency of discussing sunscreen with customers [*r_s_* (3) = 0.585, *P* < 0.003] (Figure 2). A positive correlation with ‘considering talking to customers about sunscreen’ was found [*r_s_* (3) = 0.466, *P* < 0.03]. A statistically significant negative correlation was found between wearing sunscreen and considering selling sunscreen [*r_s_* (3) = −0.471, *P* < 0.02]. No significant association was found with self-application of sunscreen and applying sunscreen to customers [*r_s_* (3) = −0.012, *P* < 0.96], or with considering applying sunscreen to customers [*r_s_* (3) = −0.100, *P* < 0.65].

**Figure 2:**
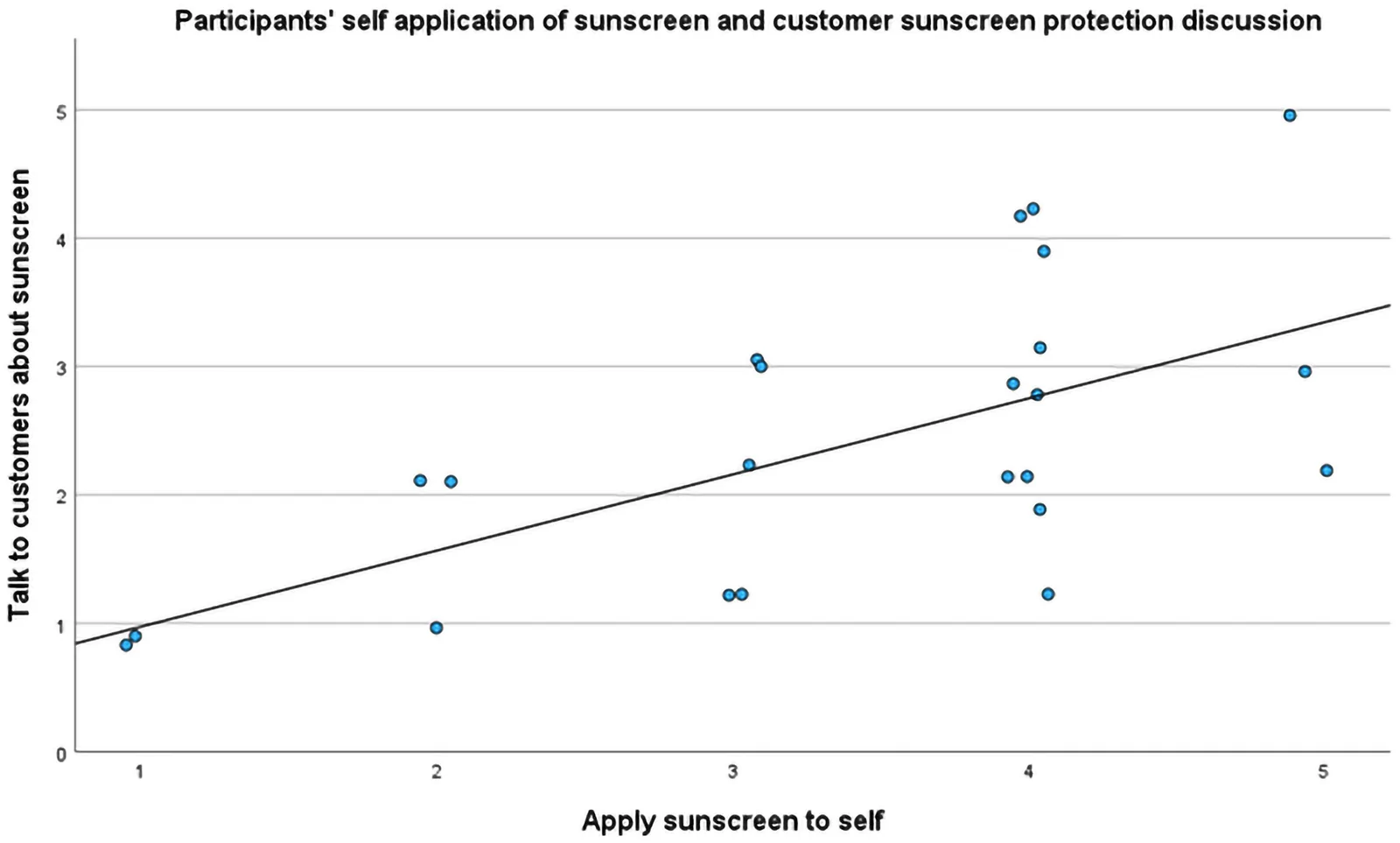
Participants’ self-application of sunscreen and frequency of discussing sunscreen with customers.

### Talking to customers about sunscreen use

Thinking sunscreen was important was found to have a statistically significant positive correlation with talking to customers about sunscreen [*r_s_* (3) = 0.463, *P* < 0.03] and considering talking to customers about sunscreen [*r_s_* (3) = 0.467, *P* < 0.03]. A statistically ­significant negative correlation was found between thinking sunscreen was important and considering selling sunscreen [*r_s_* (3) = −0.430, *P* < 0.04]. There was no significant correlation with applying sunscreen to customers [*r_s_* (3) = 0.260, *P* < 0.23] or considering applying sunscreen to customers [*r_s_* (3) = −0.157, *P* < 0.47].

### Age and time in occupation and customer sun-protection behaviour

Spearman’s rank correlation was used to identify any significant association between participants’ age or duration of time in their occupation, and their customers’ sun-protection behaviours. Older age of participants was found to have a statistically significant positive association with talking to customers about sunscreen [*r_s_* (21) = 0.473, *P* < 0.02]. No significant association was found between the age of the participants and considering talking to customers about sunscreen use [*r_s_* (21) = 0.004, *P* < 0.98], applying sunscreen to customers [*r_s_* (21) = 0.052, *P* < 0.81], considering applying sunscreen to customers [*r_s_* (21) = 0.052, *P* < 0.81] or considering selling sunscreen [*r_s_* (21) = −0.029, *P* < 0.90].

### Time in occupation and customer skin cancer prevention behaviours

Analysis of duration in their occupation as a barber and customer skin cancer prevention behaviours found no statistically significant relationship with talking to customers about sunscreen [*r_s_* (21) = 0.209, *P* < 0.34], considering talking to customers about sunscreen [*r_s_* (21) = −0.021, *P* < 0.93], applying sunscreen [*r_s_* (21) = 0.188, *P* < 0.39], considering applying sunscreen to customers [*r_s_* (21) = 0.128, *P* < 0.56] or considering selling sunscreen [*r_s_* (21) = 0.038, *P* < 0.86].

### Ethnicity and sex as influencing factors

Analysis of ethnicity as an influencing factor was not undertaken because of the 20 participants who provided their ethnicity, 80% (*n* = 16) predominantly identified as either British or English; therefore, the small sample sizes meant statistical analyses would not be meaningful. Analysis of sex as an influencing factor was also not undertaken as the participant group was predominantly male (*n* = 21; 91%); therefore, the small sample sizes meant statistical analyses would not be meaningful.

### Knowing someone who previously had skin cancer and skin cancer prevention practices

Pearson’s χ^2^ test was used to investigate any association between knowing someone who has had skin cancer and the skin cancer prevention practices of the customers of the participants. There was no significant association with knowing someone who previously had skin cancer and talking to customers about sunscreen [χ^2^ = 13.36, degrees of freedom (df) = 8, *P* < 0.10], considering talking to customers about sunscreen (χ^2^ = 14.96, df = 8, *P* < 0.06), applying sunscreen to customers (χ^2^ = 0.18, df = 2, *P* < 0.92) or considering applying sunscreen to customers (χ^2^ = 10.31, df = 8, *P* < 0.24).

Those who did not know someone who had had skin cancer were statistically significantly more likely to consider selling sunscreen (χ^2^ = 31.58, df = 10, *P* < 0.001). Seventy-five per cent (*n* = 6) of the eight participants who would consider selling sunscreen did not know someone who has had skin cancer.

### Barbers’ opinions on providing advice and sunscreen protection to customers

Qualitative responses from participants included some insightful comments (Table 4). One response was significant in identifying a potential contributing factor to the rise in male head-and-neck skin cancers:

‘When I started in this trade hair styles were long in general and hair was a natural protection against the sun, since the 1990s hair has got shorter and shorter and now men are wearing their hair shorter than I have ever seen it in all of my years, even if hair is worn with say 2 cm of hair the sun’s rays will still get through the hair, and burn the scalp.’

Completing the survey appeared to have inspired another participant:

‘Very enlightening on the subject after completing I have had thoughts to create hair products for men with UV protection.’

Most participants’ comments revealed a willingness to discuss sunscreen protection with customers:

‘Some time I will tell customers with short hair to be careful in the sun and apply sun cream. Although we don’t discuss this often, we’d be happy to in order to raise more awareness.’

**Table 4:** Barbers’ opinions on providing advice and sunscreen protection to their customers

Do you have any thoughts on this topic that you would like to share?
‘I hadn’t thought about it until now. I can use sunscreen for a client from now on.’
‘Very enlightening on the subject after completing I have had thoughts to create hair products for men with UV protection.’
‘Some time I will tell customers with short hair to be careful in the sun and apply sun cream. Although we don't discuss this often, we’d be happy to in order to raise more awareness.’
‘I am very aware of protecting skin.’
‘I am a male barber of many years and see a lot of skin cancer damage to my more elderly clients who never had the knowledge of the sun’s damage in their early years. I myself am bald so regularly apply SPF 50+ in the sun and do talk about the sun’s harm to exposed skin.’
‘We do monitor customer’s moles, recommend doctor visit if they look suspicious. We all keep notes and photos to help us monitor skin conditions. We are also thinking of investing in getting a Woods lamp.’^a^
‘I feel it is a barber’s duty to discuss and check for moles or any signs of skin cancer on the scalp, the same as any other scalp conditions such as ring worm and what not, but in the current climate adding expenses like suncream onto our products is an expense we don’t need. We can recommend to slap it on and that seems a good way to start.’
‘Having been in the trade since 1969, I have seen many changes in styles over the years. When I started in this trade hair styles were long in general and hair was a natural protection against the sun, since the 1990s hair has got shorter and shorter and now men are wearing their hair shorter than I have ever seen it in all of my years, even if hair is worn with say 2 cm of hair the sun’s rays will still get through the hair, and burn the scalp, this is a fashion and may change in time, it is strange that British men seem to think that having your hair short in a hot spell cools you down where if anything the opposite applies. Sunscreen applied everyday would obviously help but how many men would apply it that often.’
‘I don’t think any brands of hair products for men have a sunscreen in their range. We stock an Australian company’s products, and they don’t have one. This might suggest no one is discussing it.’
‘Need more SPF product inclusion. General sunscreen knowledge needs to be spread. I for one am very pale and fair skinned so I am more conscious of applying. However, I do see a lot of males that wouldn’t apply sunscreen let alone on their head. I am a new barber in the hair industry and would welcome any new research feedback or even SPF products to have these conversations with all clients.’

SPF, sun protection factor; UV, ultraviolet.

^a^A Wood’s lamp projects UV light onto the skin to help detect dermatological conditions, including fungal, bacterial and malignant skin conditions.^24^

## Discussion

The results of this survey of barbers in a UK coastal city found that over half were aware of head-and-neck sunscreen products, regularly used them and believed sun protection was very important; however, <20% regularly discussed sunscreen with customers, <10% had ever applied sunscreen to a customer and none of the participating barbershops sold sunscreen.

Participants’ future skin cancer prevention intentions were more encouraging. Over half of barbers reported they would be somewhat or very likely to consider talking to customers about using sunscreen after a short haircut, while over a quarter would consider applying it to their customers. However, only 22% believed that their customers would want sunscreen applied to them; this assumption could warrant further investigation. Notably, more than one-third of participants reported they would consider selling sunscreen in their barbershop, which could positively influence uptake through increased availability and promotion.

Barbers’ personal sun-protection behaviours were positively associated with their customers’ skin cancer protection practices. Participants who regularly applied sunscreen to themselves and believed it was important were more likely to engage in sunscreen discussions with customers and to consider such conversations in the future. All participants who reported regular personal sunscreen use regularly discussed sunscreen with customers. However, given these results, a surprising finding was that most participants who would consider selling sunscreen reported rarely or never wearing sunscreen.

Age was found to influence talking to customers about sunscreen. Participants who frequently or always discussed sunscreen with customers had a higher mean age (48.5 years) than the overall group average of 41.0 years. Older participants were also more likely to know someone who had experienced skin cancer, with this group having a mean age of 49 years. Notably, all participants who frequently or always talked to customers about sunscreen reported knowing someone who had previously had skin cancer. Interestingly, the length of time participants had worked as barbers was not found to influence customer skin-­cancer protection practices.

Another unexpected finding was that barbers who reported not knowing someone affected by skin cancer were more likely to consider selling sunscreen than those who did. All participants who recorded knowing someone who had had skin cancer identified as either British or English.

Despite current low engagement in customer skin cancer prevention practices, the open-ended responses participants provided suggested this is down to lack of consideration rather than resistance to providing this service. However, two participants expressed doubts regarding customers’ willingness to wear sunscreen.

Although only 26% would consider applying sunscreen to customers, concerns about product cost and assumptions about customer interest were identified in the open-ended question as potential barriers. Further research into customer attitudes could help determine the feasibility of providing this service.

Studies have shown that most men in the UK do not regularly wear sunscreen,^8,10^ with some believing the sun is not strong enough to burn them and others simply not thinking about wearing sunscreen. Among men who routinely visit a barber, there is an opportunity for barbers to discuss head-and-neck skin protection and create awareness of the importance of wearing sunscreen in this area, particularly after a short haircut. Encouraging barbershops to sell sunscreen products could also create an association for customers with wearing sunscreen alongside the convenience of being able to easily purchase these products.

The findings of this study are encouraging; however, they may not be representative of other areas of the UK. Furthermore, we recognize the limitations of this small pilot study undertaken to determine a sample group of UK barbers’ personal and customer skin cancer protection behaviours. A larger sample size would add validity to these findings; however, with no comparable studies in the UK or other countries we believe that, despite the small sample size, the findings of this study still have merit.

Further research on larger sample groups is needed to validate these findings and to evaluate the efficacy of targeted interventions such as providing skin cancer prevention and awareness training to barbers and encouraging them to sell sunscreen products. Resources exist to train hair professionals in skin cancer awareness and some, such as the Karen Clifford skin cancer charity (SKcin),^25^ include sun protection. However, these courses are not free and uptake from barbers has been poor. There is a need for a free prevention and detection resource that is easily accessible and inclusive.

This study identifies a promising opportunity for improving skin cancer prevention practices among men through engaging with their barber. Barbershops represent a familiar and trusted community setting where preventative health messages, such as head-and-neck sun protection, could be effectively communicated to those who are less likely to interact with conventional health promotion channels. Although no previous UK-based studies have specifically explored barbers’ roles in skin cancer prevention, previous research has indicated that barbers are interested in gaining further training in skin cancer awareness.^19^

There is a critical need to develop a national skin cancer prevention strategy that prioritizes education and early detection alongside treatment. Public awareness of UV skin damage risks, particularly among men who underestimate the danger of sun exposure in the UK, must be improved. Using barbers as health advocates could offer a cost-effective approach to reinforcing skin cancer prevention behaviours and reducing the future burden of skin cancer treatment on individuals and treatment cost to the NHS.
